# Hyperglycemia Alters Astrocyte Metabolism and Inhibits Astrocyte Proliferation

**DOI:** 10.14336/AD.2017.1208

**Published:** 2018-08-01

**Authors:** Wenjun Li, Gourav Roy Choudhury, Ali Winters, Jude Prah, Wenping Lin, Ran Liu, Shao-Hua Yang

**Affiliations:** ^1^Department of Pharmacology and Neuroscience, University of North Texas Health Science Center, Fort Worth, TX 76107, USA; ^2^Department of Orthopedic Surgery, The Second Affiliated Hospital, Fujian Medical University, Fujian Province, 362000, China

**Keywords:** diabetes, astrocyte, metabolism, AMP-activated protein kinase

## Abstract

Diabetes milieu is a complex metabolic disease that has been known to associate with high risk of various neurological disorders. Hyperglycemia in diabetes could dramatically increase neuronal glucose levels which leads to neuronal damage, a phenomenon referred to as glucose neurotoxicity. On the other hand, the impact of hyperglycemia on astrocytes has been less explored. Astrocytes play important roles in brain energy metabolism through neuron-astrocyte coupling. As the component of blood brain barrier, glucose might be primarily transported into astrocytes, hence, impose direct impact on astrocyte metabolism and function. In the present study, we determined the effect of high glucose on the energy metabolism and function of primary astrocytes. Hyperglycemia level glucose (25 mM) induced cell cycle arrest and inhibited proliferation and migration of primary astrocytes. Consistently, high glucose decreased cyclin D1 and D3 expression. High glucose enhanced glycolytic metabolism, increased ATP and glycogen content in primary astrocytes. In addition, high glucose activated AMP-activated protein kinase (AMPK) signaling pathway in astrocytes. In summary, our *in vitro* study indicated that hyperglycemia might impact astrocyte energy metabolism and function phenotype. Our study provides a potential mechanism which may underlie the diabetic cerebral neuropathy and warrant further *in vivo* study to determine the effect of hyperglycemia on astrocyte metabolism and function.

Diabetes milieu is a complex metabolic disease that has been known to associate with high risk of various neurological disorders. Several lines of evidence suggest that hyperglycemia can lead to progressive functional and structural abnormalities in the brain. Studies derived from rodent models have indicated that diabetes impairs hippocampal LTP and causes cognitive function impairments [1-3]. Diabetic rats showed reduced dendrites and synaptic spines, which may be associated with decreased BDNF expression [4, 5]. Diabetes increases brain damage after ischemic stroke [6, 7]. Type 2 diabetes (T2DM) is associated with global brain atrophy and an increased burden of small-vessel disease [8]. There is an increasing clinical and epidemiological evidence support the link between diabetes and Alzheimer disease (AD) [9].

The brain is by far the most expensive organ in term of energy expenditure in the whole body with a strict dependence on glucose. In the brain, neurons have a constantly high glucose demand which depends on the extracellular concentration of glucose [10]. Hyperglycemia in diabetes could dramatically increase neuronal glucose levels which leads to neuronal damage, a phenomenon referred to as glucose neurotoxicity [10, 11]. Several mechanisms have been proposed to underlie the glucose neurotoxicity, including glucose-driven oxidative stress and protein glycation. In addition, indirect effects through accessory glia might be involved in the glucose neurotoxicity [10].

Astrocytes are the most abundant glial cells in the CNS and play important roles in maintaining normal brain functions, including physical support, neurotransmitter uptake, energy storage and supply, blood-brain barrier formation, cerebral blood flow and synaptic transmission regulation [12-15]. Astrocytes response to all forms of CNS insult by a process commonly referred as reactive astrogliosis [15]. There is increasing evidence that astrocytes might be also involved in the diabetic cerebral neuropathy. High glucose increased reactive oxygen species (ROS) production, inflammatory cytokines expression, and cell apoptosis in primary astrocytes [16, 17]. Prolonged high glucose treatment caused irreversible impairment of gap junction communication among astrocytes [18]. Furthermore, change of glial fibrillary acidic protein (GFAP) expression has been found in rodent diabetes model [19, 20]. Anatomically glucose might be primarily transported into astrocyte given that astrocyte is the component of blood brain barrier (BBB) [21, 22]. We speculated that hyperglycemia may have more direct impact on astrocyte metabolism and function. Surprisingly, most the studies using primary astrocyte culture have been using hyperglycemia level glucose and the effect of high glucose on astrocyte metabolism and function is not clear. In the current study, we determined the effect of hyperglycemia on astrocyte metabolism and function.

## MATERIALS AND METHODS

### Cells and Reagents

Primary astrocytes were prepared according to previous study with modifications [23, 24]. Briefly, cortex was dissected from postnatal day 0-1 C57BL/6 mouse pups. Cells were seeding in normal glucose Dulbecco’s Modified Eagle Medium (DMEM) with 10% Fetal Bovine Serum (FBS). Cells were then cultured CO2 incubators with medium changed every 2-3 days. When cells became confluent, plate was shaken on an orbital shaker. Then, medium was removed, and cells were split into new plates and culture for 2-7 days. These cells were then seeded and used for experiments. Propidium iodide (PI), annexin V, rotenone, carbonyl cyanide-4-(trifluoromethoxy) phenylhydrazone (FCCP), and oligomycin were purchased from Sigma. Anti-GFAP antibody and anti-actin antibody were purchased from Santa Cruz; all the other antibodies were purchased from Cell Signaling. Quantitative real-time PCR was carried out using SYBR Green PCR Master Mix (Promega) and 7300 Real-Time PCR System from Applied Biosystems.

### Growth Curve Assay

Astrocyte were seeded into 12-well (40,000 per well) or 24-well (25,000 per well) culture plates in DMEM with pyruvate and 10% FBS. Cells were cultured for two days, and then medium was replaced with new normal glucose (5.5 mM) or high glucose (25 mM) DMEM (with pyruvate and 10% FBS). Plates were incubated in a humidified incubator at 37 °C and 5% CO_2_. Cells were harvested on each indicated day using 0.25% trypsin-EDTA (Invitrogen) and counted using an inverted phase contrast Zeiss Invertoskop microscope.

### Cell Cycle and Apoptosis Analysis

Cells were plated at a density of 50,000 per well in 24-well plate and cultured overnight. The following day, the cells were deprived of FBS overnight to standardize the cell cycle followed by fresh DMEM containing 10% FBS and the indicated concentration of glucose. At the specified times, cells were harvested using 0.5% trypsin (Invitrogen) and washed with wash buffer (0.1% FBS in PBS) twice to remove trypsin. Cells were fixed in ice-cold 70% ethanol for 45 min at 4 °C. Ethanol was removed by washing twice with PBS, and the cells were incubated with propidium iodide (PI) (40 μg/ml) and RNase (10 μg/ml) for 30 min at 37 °C. Samples were analyzed using Beckman Coulter FC500 Flow Cytometry Analyzer. Cell apoptosis was analyzed using flow cytometry (BD LSR II, San Jose, CA, USA) with annexin-V and propidium iodide staining and Fluorometric TUNEL kit purchased from Promega.

### Extracellular Flux Analysis

Astrocytes were plated at a density of 50,000/well in normal glucose DMEM medium (10% FBS) in a Seahorse XF24 plate and cultured for 24 hours before medium was replaced with new normal glucose DMEM or high glucose DMEM. One hour before assay, media were exchanged for XF24 media with 5.5 mM glucose or 25 mM glucose. Rotenone, FCCP, and oligomycin were diluted into XF24 media and loaded into the accompanying cartridge to achieve final concentrations of 2 μM, 1 μM, and 1 μg/ml, respectively. Injections of the drugs into the medium occurred at the time points specified. Oxygen consumption rate (OCR) and extracellular acidification rate (ECAR) were monitored using a Seahorse Bioscience XF24 Extracellular Flux Analyzer. Each cycle was set as: mix for 3 minutes, delay for 2 minutes and then measure for 3 minutes.

**Figure 1.**
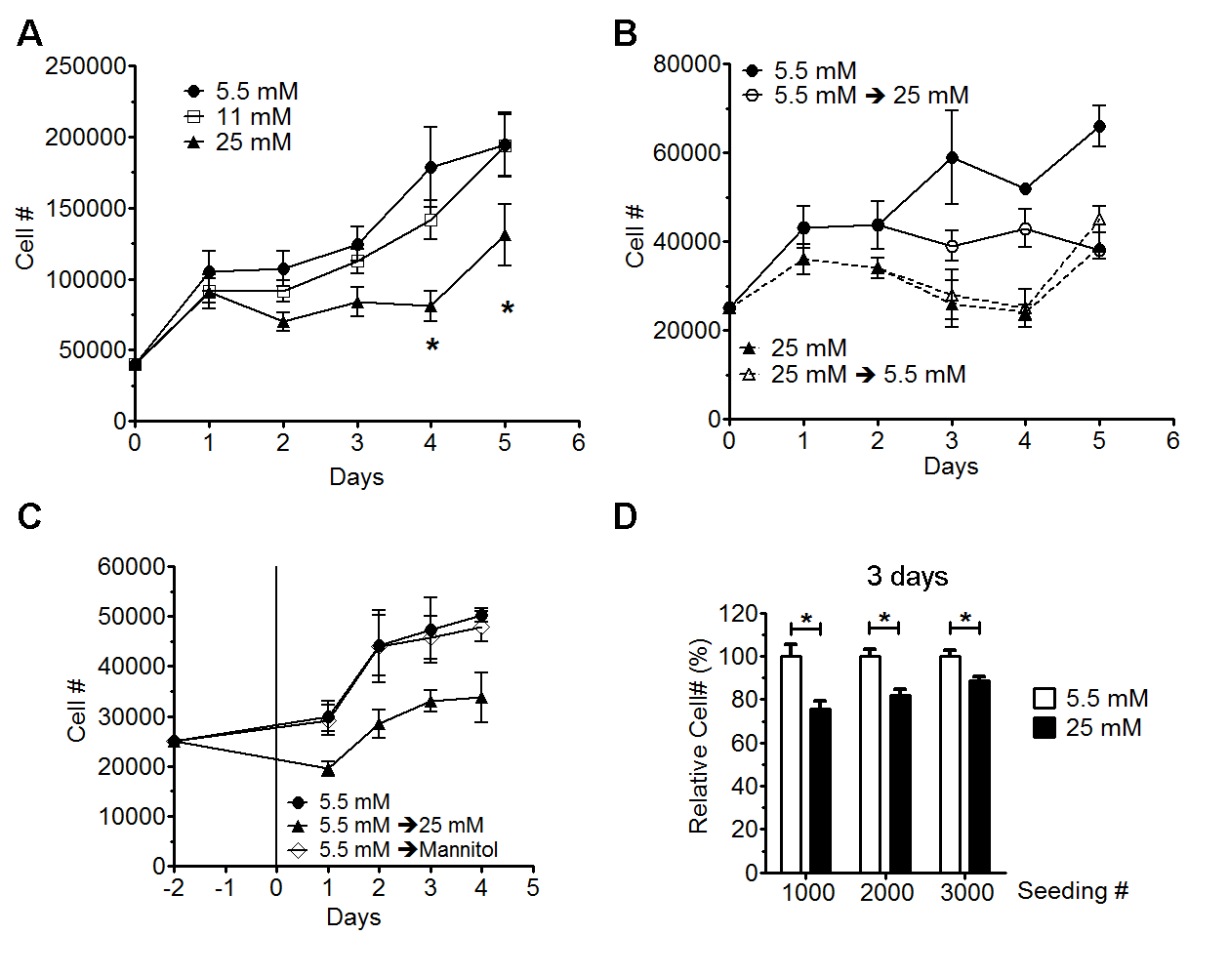
High glucose inhibited astrocyte proliferation **A**) Growth curve assay of primary astrocytes cultured in normal (5.5 mM) and high glucose (11 or 25 mM) for 2 weeks. 40,000 cells per well were seeded in 6-well plates (n=6) and cells were counted at 1 to 5 days after culture at each condition. High glucose at 25 mM significantly inhibited primary astrocyte proliferation (* p < 0.05, n=6). **B**) Growth curve of astrocytes with medium switched on day 2 (n=3-6). Switch normal glucose medium (5.5 mM) dramatically inhibited astrocyte proliferation. Switch high glucose medium (25 mM) to normal glucose medium had no impact on astrocyte proliferation. **C**) Normal glucose cultured astrocytes were seeded and cultured for 2 days before medium was replaced with normal glucose medium (5.5 mM), normal glucose medium with mannitol (Mannitol), and high glucose medium (25 mM) (n=4). Osmolarity adjusted by mannitol has no impact on growth curve assay of primary astrocyte culture. **D**) Normal (5.5 mM) and high glucose (25 mM) cultured astrocytes were seeded in 96-well plates and cultured for 3 days and viability was analyzed by calcein AM. High glucose significant inhibited astrocyte proliferation (* p<0.05, n=10).

### ATP Assay

ATP kit was purchased from Invitrogen. Astrocytes were seeded into 24-well plates at a density of 50,000 cells/well and incubated at 37 °C and 5% CO2. For ATP assay, cells washed with PBS twice and lysed with 100 μl of ATP assay buffer (500 mm Tricine buffer, pH 7.8, 100 mm MgSO4, 2 mm EDTA, and 2 mm sodium azide) containing 1% Triton X-100. 10-μl cell lysate was added in triplicate to a white 96-well plate along with an ATP standard curve. Before reading the plate, 100 μl of ATP reaction buffer (30 μg/ml d-luciferin, 20 μm DTT, and 25 μg/ml Luciferase) was added to each well. Luminescence was measured using a Tecan Infinite F200 plate reader. Protein concentration was measured simultaneously using the Pierce 660 nm Protein Assay (660 nm absorbance), and ATP production was normalized to protein content of the samples.

### Glycogen assay

Glycogen level was measured using a Glycogen Assay Kit (Sigma-Aldrich, St. Louis, MO) following the manufacturer’s manual. Glycogen level was normalized to protein concentration of each sample.

### Scratch Assay

The astrocytes (2.5 × 105 cells/well) were cultured in normal glucose (5.5 mM) and high glucose (25 mM) DMEM (10% FBS) to a monoconfluent layer in 6-well cell culture plates. Using sterile 200 µl pipette tip scratches were made on the cell layer, the plates were then rinsed with sterile PBS to remove cell debris and replaced with fresh cell culture media. At 0, 24 and 48 hrs after scratch, cells were stained with Calcein AM (10 µM), fluorescent images were obtained randomly using a Zeiss fluorescence microscope.

**Figure 2.**
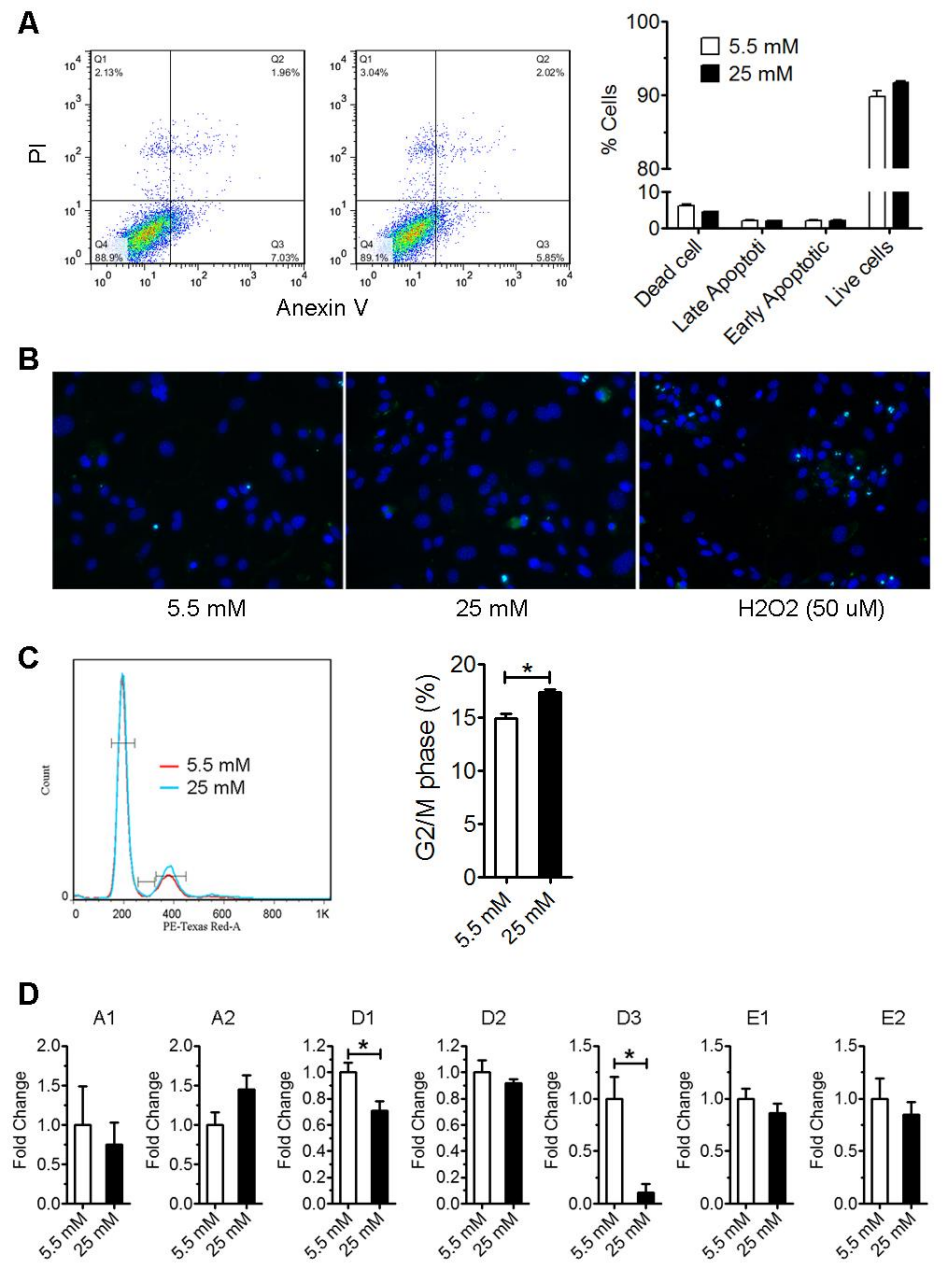
High glucose induced astrocyte cell cycle arrest without increasing apoptosis **A**) Flow cytometry analysis of Annexin-V and PI staining of astrocytes cultured in normal (5.5 mM) and high glucose (25 mM) medium for 3 days (n=4). **B**) TUNEL staining (green) of astrocytes cultured in normal (5.5 mM) and high glucose (25 mM) for 3 days. Astrocytes were treated with 50 μM H_2_O_2_ for ~12 hours as positive control. Cells were counter stained with DAPI (blue). **C**) Astrocytes were cultured in normal (5.5 mM) and high glucose (25 mM) for 3 days. Then astrocytes were stained with PI and analysis by flow cytometry (* p<0.05 vs 5.5 mM, n=4). **D**) Real-time rtPCR analysis of cyclin expression in astrocytes cultured in normal (5.5 mM) and high glucose (25 mM) medium for 2 days (* p<0.05 vs 5.5 mM, n=4).

### Statistical Analysis

All data are presented as the mean ± S.E.M. The significance of differences among groups with one independent variable was determined by one-way ANOVA with a Tukey’s multiple comparisons test for planned comparisons between groups when significance was detected. The significance of differences among groups where two independent variables were present was determined by two-way ANOVA with a Bonferroni post-test for planned comparisons between groups when significance was detected. For all tests, p < 0.05 (*) was considered significant.

**Figure 3.**
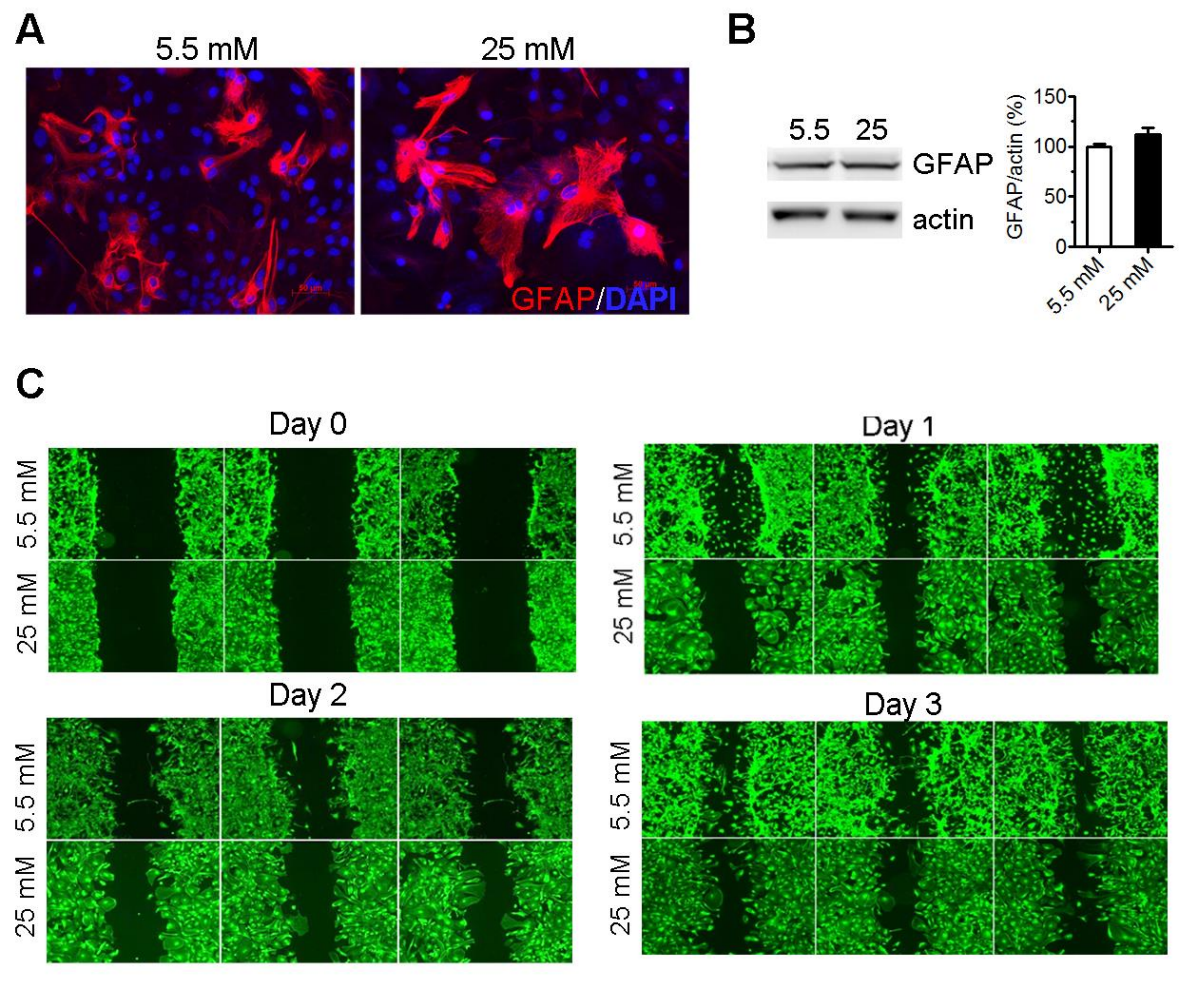
High glucose altered astrocyte morphology and inhibited cell migration **A**) GFAP staining of astrocytes cultured in normal (5.5 mM) and high glucose (25 mM) for 3 days. Nucleus was stained by DAPI. **B**) Western blot analysis of GFAP expression in astrocytes cultured in normal (5.5 mM) and high glucose (25 mM) for 2 weeks (n=4). **C**) Astrocytes were maintained in normal (5.5 mM) and high glucose (25 mM) medium for 2 weeks and then seeded for scratch assay. Cells were staining with calcein AM and fluorescent images were obtained.

## RESULTS

### High glucose inhibited astrocyte proliferation

Primary astrocytes were maintained in DMEM media (10% FBS) with 5.5, 11 or 25 mM glucose for a week, and cells were then split and cultured for 3-5 days before seeding for growth curve analysis. Forty thousand cells were seeded in 6-well plates and cultured in media with 5.5, 11 and 25 mM glucose. Primary astrocytes cultured in 25 mM glucose proliferated significantly slower than astrocytes cultured in 5.5 and 11 mM glucose (Fig. 1A). There was no significant difference in the growth rates of astrocytes cultured 5.5 and 11 mM glucose. To determine whether the effect of high glucose on astrocyte proliferation was reversible, astrocytes were seeded at a density of 25,000 per well in 12-well plates and maintained in 5 or 25 mM glucose. At 2 days after seeding, 5.5 and 25 mM glucose were switched to 25 or 5.5 mM, respectively. As expected, astrocyte proliferation was significantly inhibited when glucose was switched from 5.5 to 25 mM. Interestingly, no change of proliferation rate was observed after switching glucose from 25 to 5.5 mM, suggesting that high glucose (25 mM) caused an irreversible inhibition of astrocyte proliferation (Fig. 1B). Osmolarity was 333 mOsm/L for DMEM with 5.5 mM glucose (normal glucose DMEM) and 336 mOsm/L for DMEM with 25 mM glucose (high glucose DMEM), indicating that difference in osmolarity was unlikely responsible for the inhibition of astrocyte proliferation induced by high glucose. Indeed, adjusting of osmolarity by addition of 19.5 mM mannitol to 5.5 mM glucose media did not inhibit astrocyte proliferation (Fig. 1C).

**Figure 4.**
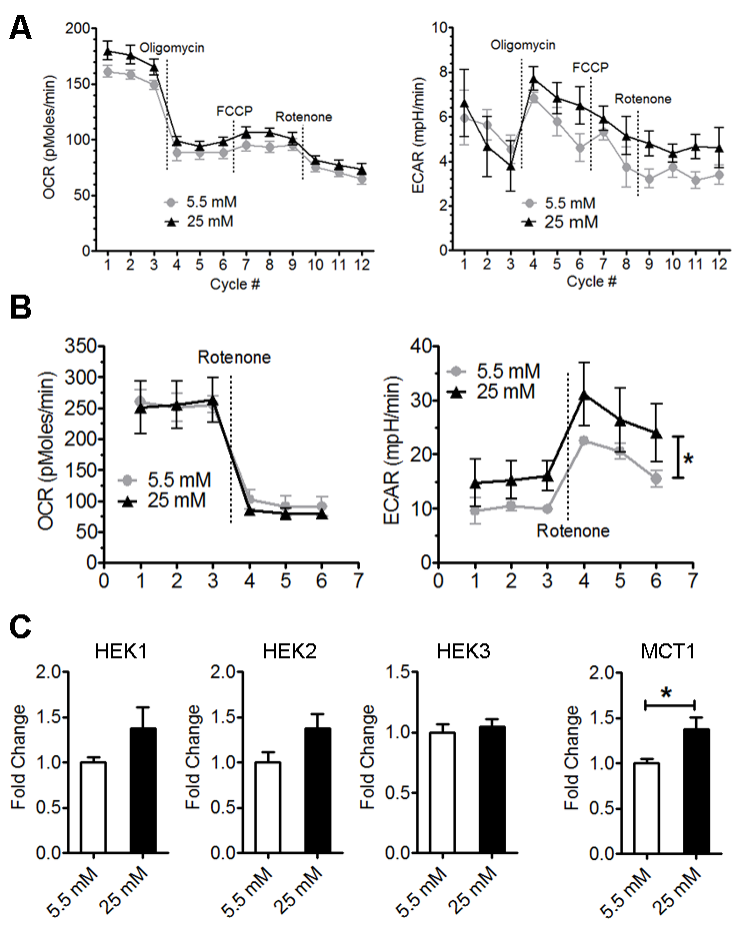
High glucose enhanced astrocyte glycolysis Seahorse extracellular flux analysis of oxygen consumption rate (OCR) and extracellular acidification rate (ECAR) after 6-hour (**A**) and 24-hour culture (**B**) in normal (5.5 mM) and high glucose (25 mM) (* p<0.05 vs 5.5 mM, n=5-6). **C**) Real-time rtPCR analysis of HEKs and MCT1 expression of astrocytes after 24-hour culture in normal (5.5 mM) and high glucose (25 mM) (* p<0.05 vs 5.5 mM, n=6-8).

The effect of high glucose on astrocyte proliferation was further confirmed by calcein AM assay. Astrocytes were seeded in 96-well plates at densities of 1,000, 2,000, and 3,000 cells per well in normal glucose (5.5 mM) DMEM. Cells were allowed to attach for 48 hours before medium was replaced with new normal glucose DMEM or high glucose DMEM. After culturing for 3 days, cells were stained with calcein AM and cell viability was measured by a fluorescence plate reader. Astrocyte viability cultured in high glucose (25 mM) was significant decreased compared with astrocytes cultured in normal glucose (5.5 mM) (Fig. 1D).

### High glucose induced cell cycle arrest without increasing apoptosis

We speculated that the reduced cell viability/number by high glucose could be attributed to the reduction of cell proliferation and/or increase of cell death. Flow cytometer of annexin V and PI staining of astrocytes after 3-day culture in high glucose DMEM showed not demonstrated that high glucose (25 mM) did not impact cell death (Fig. 2A). Consistently, terminal deoxynucleotidyl transferase dUTP nick end labeling (TUNEL) assay suggested that there was no difference in astrocyte apoptosis after 3-day culture in high glucose DMEM (Fig. 2B). Cell cycle analysis found that high glucose (25 mM) increased cells at the G2/M phase, suggesting an arrest of cell cycle in G2/M phase (Fig. 2C). Real-time PCR indicated that cyclin D1 and D3 expression were significantly decreased after 48-hour culture in high glucose medium, while cyclin A1, A2, D2, E1 and E2 expression were not significantly changed (Fig. 2D). Taken together, these data suggest that high glucose (25 mM) inhibited astrocyte proliferation through induction of cell cycle arrest at G2/M phase.

### High glucose altered astrocyte morphology and inhibited cell migration

Previous study has shown that high glucose treatment could change the morphology of retinal astrocytes [17]. We found that astrocytes cultured in high glucose DMEM showed a “flat” and “spread out” morphology with fewer processes compared with astrocytes cultured in normal glucose DMEM (Fig. 3A, C). None significant change in GFAP expression was found in astrocyte after prolonged culture in high glucose DMEM (Fig. 3B). Scratch assay indicated that high glucose inhibited astrocytes migration (Fig. 3C).

**Figure 5.**
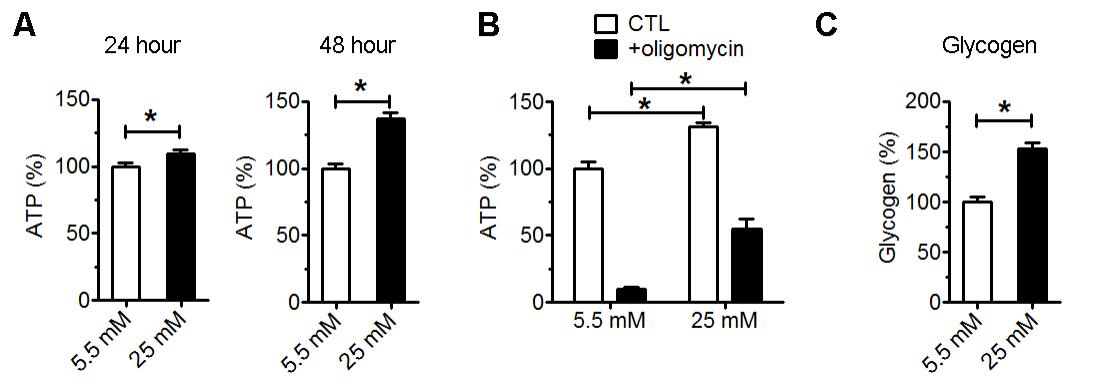
High glucose increased ATP content and glycogen storage **A**) Total ATP level of astrocytes after 24- and 48-hour culture in normal (5.5 mM) and high glucose (25 mM) (*p<0.05 vs 5.5 mM, n=4). **B**) Cellular ATP level of astrocytes after 72-hour culture in normal (5.5 mM) and high glucose (25 mM). Oligomycin was added to astrocyte culture 2 hours before ATP assay (*p<0.05 vs 5.5 mM, n=3). **C**) Glycogen assay of astrocytes cultured for 72 hours in normal (5.5 mM) and high glucose (25 mM) (*p<0.05 vs 5.5 mM, n=4).

### High glucose increased astrocyte glycolysis

The effect of high glucose on astrocyte metabolism was examined using a Seahorse XF-24 extracellular flux analyzer. No significant change in the oxygen consumption rate (OCR) or extracellular acidification rate (ECAR) was observed in astrocyte after 6-hour culture in high glucose DMEM (Fig. 4A). After 24-hour treatment, ECAR was significantly increased in high glucose astrocyte culture, suggesting increased lactate production from glycolysis (Fig. 4B). We determined the effect of high glucose on the expression of hexokinases (HEK1, HEK2 and HEK3), which catalyze the phosphorylation of glucose in glycolysis, and astrocyte lactate transporter monocarboxylate transporter 1 (MCT1), using real-time PCR. We found that MCT1 mRNA level was significantly increased by high glucose culture (Figure 4C), and a trend of increase of HEK1 and HEK2 mRNA level in high glucose culture.

**Figure 6.**
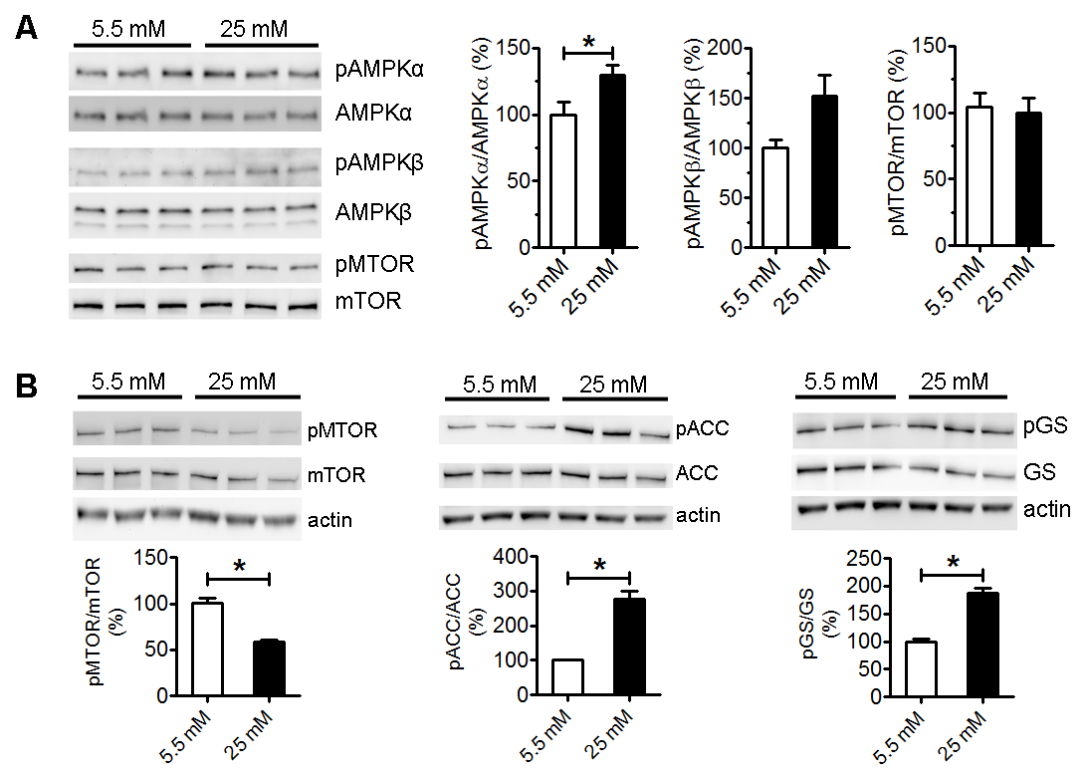
High glucose activated AMPK pathway **A**) Western blots and statistical analysis of AMPK and mTOR pathway in astrocytes after 4-day culture in normal (5.5 mM) and high glucose (25 mM) (*p<0.05 vs 5.5 mM, n=3). **B**) Western blot and statistical analysis of mTOR, ACC and GS activation after 2-week culture in normal (5.5 mM) and high glucose (25 mM) (*p<0.05 vs 5.5 mM, n=3).

### High glucose increased ATP content and glycogen storage

We determined the effect of high glucose on ATP levels in the astrocytes. High glucose significantly increased total ATP content after 24 and 48-hour culture (Fig. 5A, B). Interestingly, about 90% reduction of ATP content was observed in normal glucose cultured astrocyte upon 2-hour oligomycin (ATP synthase inhibitor) treatment. On the other hand, only about 50% reduction of ATP in astrocyte cultured in high glucose, suggesting that the increase of ATP production was mainly from glycolysis pathway (Fig. 5B). Furthermore, increase of glycogen content was increased in high glucose cultured astrocytes (Fig. 5C).

### High glucose increased AMPK activation

The AMPK pathway is a key cellular nutrient and energy sensor/regulator. AMPK activation was significantly increased in primary astrocyte after 4-day high glucose culture as compared with normal glucose culture (Fig. 6A). While mTOR activation was not changed at 4 days after high glucose culture, prolonged high glucose culture for 2 weeks significantly decreased phosphorylation of mTOR and increased phosphorylation of Acetyl-CoA carboxylase (ACC) and glycogen synthase (GS) (Fig. 6B). Taken together, these data indicated that astrocytes alter their metabolism to high glucose environment by enhancing glycolysis and glycogenesis and increase AMPK signaling pathway.

## DISCUSSION

Mammalian brain is characterized by high metabolism of glucose which is transported into the brain through insulin-independent glucose transporter protein 1 and 3 (GLUT-1 and GLUT-3). As a consequence, a linear relationship between plasma glucose concentrations and brain glucose content occurs over a wide range from hypoglycemia to hyperglycemia [25]. There is increasing evidence that astrocyte has higher glucose metabolism as compared with neurons and that activation of somatosensory cortex enhances glucose uptake predominantly in astrocyte [26]. In the current study, we found that high glucose (25 mM) induced dramatic impact on astrocyte phenotype *in vitro*. High glucose irreversibly inhibited astrocyte proliferation likely mediated by cell cycle arrest without impacting apoptosis. Astrocyte proliferation is an important component of reactive gliosis in response to various brain damage [27, 28]. Diabetes has been shown to inhibit astrocyte activation after ischemic stroke [29]. Our data suggests that inhibition of astrogliosis in diabetes might be attributed to the inhibitive action of high glucose on astrocyte proliferation.

Whereas neurons possess highly oxidative metabolism, astrocyte has unique metabolic phenotype that rely more on glycolytic metabolism. Our study indicated that high glucose did not impact oxidative phosphorylation. On the other hand, high glucose enhanced glycolysis and increased lactate production and ATP content in astrocytes. Increased lactate level has been found in retina and brain of diabetic rats [30, 31]. Consistently, increased brain lactate concentration has been found in diabetic patients [32]. In the last decade, our understanding of neuroenergetics is rapidly evolving from the “neurocentric” view to an integrated picture of neuron-astrocyte coupling [33, 34]. Studies in recent years have indicated that astrocyte-neuron lactate shuttle provides substrate supply for neuron metabolism [34]. We also found that high glucose exposure increased MCT1 expression, which transports lactate produced in astrocytes to the extracellular space, where it can be up-taken by neurons. Energy metabolism of astrocytes is tightly coupled to that of neurons [35, 36]. While lactate can be used as an important energy source for neurons, excessive lactate may cause damage to neurons, especially under pathological condition such as traumatic brain injury (TBI) or ischemic stroke [37, 38]. We expect that the high glucose-induced metabolic phenotype change might disintegrate the astrocyte-neuron coupling, hence, lead to neuronal dysfunction.

AMPK is an evolutionarily conserved energy sensor and regulator for energy metabolism [39]. Given the effect of high glucose exposure on glucose metabolism, it might not be a surprise that high glucose will impact AMPK signaling. We discovered that high glucose exposure enhanced AMPK activation evidenced by the increasing of phosphorylation of AMPKα. Consistently, inhibitory action of high glucose on downstream mTOR and acetyl CoA carboxylase (ACC) was observed, evidenced by the reduction of the mTOR phosphorylation and increase of ACC phosphorylation, respectively. AMPK is a strong suppressor of cell proliferation through the inhibition of protein, lipid, and nucleic acid synthesis [40]. We speculated that high glucose induced AMPK activation may contributed to its inhibitive action on astrocyte proliferation. Paradoxically, the increasing of AMPK activation by high glucose was not associated with an enhancement of oxidative phosphorylation in astrocyte. In addition, the increase of phosphorylation of glycogen synthase was not correlated to a reduction of glycogen content in astrocyte. We speculate that increase of glycogen content despite of AMPK activation in the astrocytes culture was due to that high glucose culture condition provides excessive glucose for glycogenesis. Both neurons and astrocytes have the similar oxidative phosphorylation capacity, in accordance with the evidence that both cell types contain equivalent numbers of mitochondria [41, 42]. Nonetheless, astrocytes and neurons prefer different metabolic pathways in part due to the different expression patterns of key genes of energy metabolism [34]. As a result, we expect that the metabolic consequence of AMPK activation might cell type-specific. The observed action of high glucose on AMPK signaling provided further evidence that high glucose indeed affects astrocyte energy metabolism.

In summary, our *in vitro* study using primary astrocyte culture indicated that high glucose exposure changed astrocyte energy metabolism and function phenotype, evidenced by the enhancement of glycolytic metabolism, activation of AMPK signaling, and inhibition of proliferation in astrocyte. Our study provides a potential mechanism which may underlie the diabetic cerebral neuropathy and warrant further *in vivo* study to determine the effect of hyperglycemia on astrocyte metabolism and function.
